# Transesophageal echocardiography detection of air embolism during endoscopic surgery and validity of hyperbaric oxygen therapy: Case report

**DOI:** 10.1097/MD.0000000000026304

**Published:** 2021-06-11

**Authors:** Ji-ling Guo, Han-bing Wang, Hong Wang, Yue Le, Jian He, Xue-qin Zheng, Zhi-hao Zhang, Guang-rong Duan

**Affiliations:** a Guangdong Medical University. Wenming East Road No.2, Zhanjiang; b Department of Anesthesiology; c Department of Information, The First People's Hospital of Foshan, North of Ling Nan Road No. 81, Foshan, Guangdong, China.

**Keywords:** air embolism, case report, hyperbaric oxygen, transesophageal echocardiography

## Abstract

**Introduction:**

Air embolism has the potential to be serious and fatal. In this paper, we report 3 cases of air embolism associated with endoscopic medical procedures in which the patients were treated with hyperbaric oxygen immediately after diagnosis by transesophageal echocardiography. In addition, we systematically review the risk factors for air embolism, clinical presentation, treatment, and the importance of early hyperbaric oxygen therapy efficacy after recognition of air embolism.

**Patient Concerns:**

We present 3 patients with varying degrees of air embolism during endoscopic procedures, one of which was fatal, with large amounts of gas visible in the right and left heart chambers and pulmonary artery, 1 showing right heart enlargement with increased pulmonary artery pressure and tricuspid regurgitation, and 1 showing only a small amount of gas images in the heart chambers.

**Diagnoses:**

Based on E_T_CO_2_ and transesophageal echocardiography (TEE), diagnoses of air embolism were made.

**Interventions:**

The patients received symptomatic supportive therapy including CPR, 100% O_2_ ventilation, cerebral protection, hyperbaric oxygen therapy and rehabilitation.

**Outcomes:**

Air embolism can causes respiratory, circulatory and neurological dysfunction. After aggressive treatment, one of the 3 patients died, 1 had permanent visual impairment, and 1 recovered completely without comorbidities.

**Conclusions:**

While it is common for small amounts of air/air bubbles to enter the circulatory system during endoscopic procedures, life-threatening air embolism is rare. Air embolism can lead to serious consequences, including respiratory, circulatory, and neurological impairment. Therefore, early recognition of severe air embolism and prompt hyperbaric oxygen therapy are essential to avoid its serious complications.

## 1 Introduction

Air embolism is a clinical syndrome in which air enters the circulation and causes vascular embolism, resulting in respiratory, circulatory and neurological dysfunction. Although life-threatening air embolism is rare, mild air embolism is encountered commonly in clinical. Risk factors for an air embolism that have been reported are previous interventions or surgeries of the bile duct system, transhepatic portosystemic shunt, blunt or penetrating trauma to the liver, inflammation of the digestive system, postsurgical gastrointestinal fistula, and particular interventional techniques.^[1]^ Gas embolism well documented entity during operative hysteroscopy, with an incidence of 10% to 50%.^[2]^ When small amounts of air do not always produce symptoms. A large bolus of air entering the venous system can cause an air lock in the right atrium and ventricle.^[3]^ The lethal volume of air embolism in adults is 3 to 5 ml/kg. Air embolism is difficult to diagnose when the patient is under general anesthesia. Therefore, active prevention, timely diagnosis and appropriate treatment are very important. In this paper, we reported 3 life-threatening air embolism under general anesthesia in our institution.

## 2 Case report

**Figure 1:**
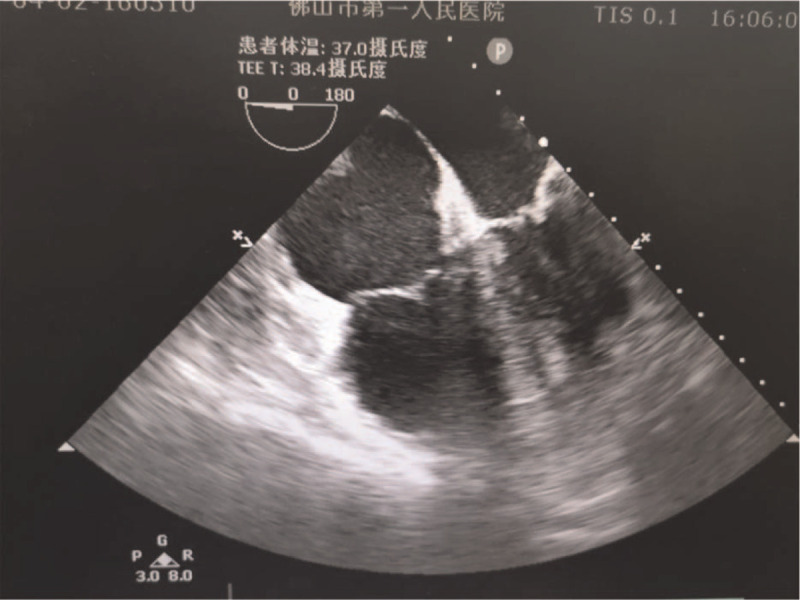
Transesophageal echocardiography showed enlarged right atrium and right ventricle with leftward deviation of the septum and mild-moderate elevation of pulmonary artery pressure, with no significant gas in the cardiac chambers.

Case 1: a 53-year-old male, 78 kg, was diagnosed with right kidney stones and scheduled for percutaneous nephrolithotripsy (PCNL). The patient had a medical history of diabetes mellitus, and his blood glucose level was well controlled by taking medicine regularly. At 14:30 on April 2, 2020, the patient underwent routine induction of intravenous anesthesia with propofol 3.5 μg/mL plasma target-controlled infusion, midazolam 2 mg, sufentanil 15 μg, and cis-atracurium 12 mg, and then intubation proceeded smoothly. The PCNL was performed in the prone position at 14:50, 8 minutes after operation, E_T_CO_2_ dropped abruptly to below 13 mmHg and the noninvasive blood pressure decrease to 86/43 mmHg, followed by an increase in blood pressure with a fluctuation range of 145 to 194/95 to 110 mmHg and heart rate fluctuating between 120 and 140 beats per minute (bpm). The patient's heart rate suddenly slowed to 40 bpm during the procedure, and the SpO_2_ fluctuated from 89% to 95%, and the Electrocardiograph (ECG) monitor showed that ST segment was extremely elevated. During this period, 25 mg urapidil was administered intermittently to lower blood pressure intravenously. The patient's blood pressure and ECG returned to normal at 15:10, the SpO_2_ was 98%, E_T_CO_2_ was 37 mmHg. Then the patient was transferred to the supine position. Arterial blood gas analysis showed that PaCO_2_ was 75 mmHg and Lac was 3.6 mmol/L at 15:15. At 15:25, transthoracic echocardiography showed suspicious gas in the cardiac chambers, at that time the patient's vital signs were normal. At 16:06, transesophageal echocardiography (TEE) showed enlarged right atrium and right ventricle with leftward deviation of the septum and mild-moderate elevation of pulmonary artery pressure, with no significant gas in the cardiac chambers (Fig. 1). At 16:35, the patient recovered spontaneous breathing and could open his eyes as commanded. After removing the tracheal tube, the patient was sent to the postanesthesia care unit for further observation. At 17:20, the patient's consciousness gradually turned into a lethargic state, and the neurology doctors were immediately requested to conduct an emergency consultation. Lung computed tomography angiography examination showed multiple exudate foci in both lungs. At 20:40, the patient's state of consciousness deteriorated further, and the patient became severely drowsy, oxygenation could not be maintained, so the patient was intubated again. Then the patient was sent to the intensive care unit for further treatment. The patient developed convulsions in the early morning of the next day and received sedation and anti-convulsive treatment. His vital signs were stable. On the first day after operation, the patient was in a coma state, with a glasgow coma score score of 3. The Lac level rose to 11.2 mmol/L. The echocardiography showed mild pulmonary hypertension with normal heart structure, and the magnetic resonance imaging showed bilateral occipitoparietal, frontal, and cerebellar hemispheres swelling, which may be induced by hypoxia. The patient's diagnosis was considered hypoxic-ischemic encephalopathy, the etiology of which remains to be defined; air embolism? The patient remained in a state of coma after conventional dehydration and cerebral protection therapy. Hyperbaric oxygen therapy (HBOT) was started on the 4th day after surgery. The patient entered the hyperbaric chamber with a tracheal tube, the hyperbaric oxygen pressure was set at 0.29 Mpa, the oxygen inhalation time was 120 minutes, the oxygen concentration in the chamber was 21.3%, the temperature was 24.2°C, and the humidity was 70.4%. After 3 treatments, the hyperbaric oxygen protocol was adjusted to a pressure of 0.25 Mpa and an oxygen inhalation time of 110 minutes, and other parameters remained unchanged. We adjusted the treatment protocol according to the condition. The glasgow coma score to 4 on the 11th postoperative day, and the patient began to respond to painful stimuli.

After 2 weeks of HBOT, the patient's bilateral corneal reflex was improved with spontaneous eye opening, and the muscle strength level of both upper limbs was grade 1, and that of both lower limbs was grade 0. After 3 weeks of HBOT, the patient's muscle strength level of both upper limbs was grade 5, the right lower limb was grade 5, and the left lower limb was grade 4, but his vision was severely impaired in both eyes, with a visual acuity of about 0.1 in both eyes. The patient received a total of 43 HBOT treatments within 2 months after surgery and was discharged with normal muscle strength levels and a return to 0.3 visual acuity in both eyes.

**Figure 2:**
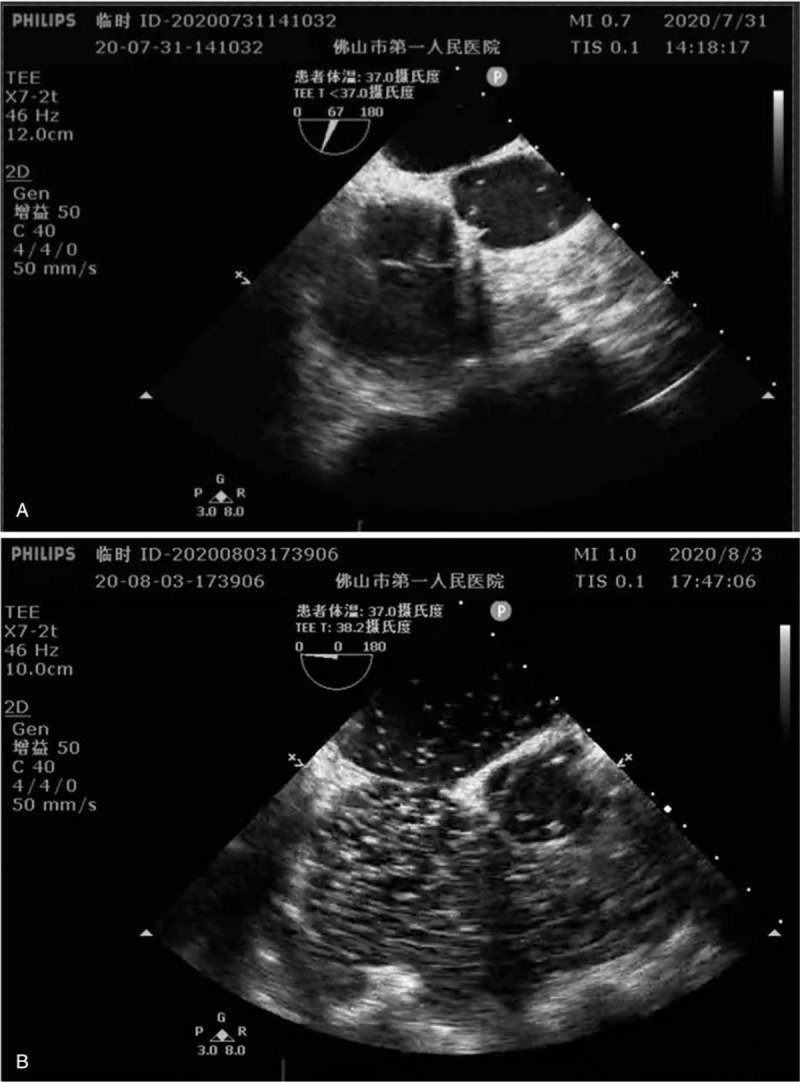
Transesophageal echocardiography demonstrated air within heart chambers.

Case 2: a 46-year-old female patient, 55 kg, was diagnosed with submucosal fibroids of the uterus, underwent hysteroscopic myomectomy under non-intubation intravenous anesthesia. The patient was in good physical condition, and preoperative examinations of the patient showed no significant abnormalities. The operation was started at 14:25 on July 31, 2020 after intravenous injection of propofol 150 mg and sufentanil5ug, with additional propofol 20 to 30 mg intermittently during the operation. At 14:40, the patient developed shortness of breath, with SpO_2_ dropped to 88%, blood pressure 107/65 mmHg, and scattered rales in both lungs, then the patient was given intravenous methylprednisolone and furosemide, general anesthesia with tracheal intubation and arterial blood gas analysis was performed. After intubation, the patient's SpO_2_ rose to 95%, with E_T_CO_2_ 14 mmHg and normal electrolytes. The ECG monitor showed low ST segment. TEE examination showed enlarged right atrium and right ventricle with small air bubbles scattered in both the right and left heart chambers (Fig. 2 A). Multidisciplinary consultation was immediately organized in the operating room, and the final definitive diagnosis of air embolism was made. At 15:20, the patient was awake and resumed spontaneous breathing with SpO_2_ of 97%. The patient was diagnosed with air embolism and was immediately given HBOT with the pressure was 0.29Mpa, the oxygen inhalation time was 120 minutes, and the other parameters were the same as in the case1. During treatment period, the patient had a transient convulsion, which improved after intravenous injection 3 mg midazolam. After HBOT, the patient's cranial computed tomography (CT) scan showed no significant abnormalities, and a repeated cardiac echocardiogram revealed no abnormalities in intracardiac structures or blood flow. On the first day after operation, the patient continued HBOT and reviewed, the pressure was 0.25Mpa, the oxygen time was 110 minutes. The CT results indicated no cerebral ischemia and hypoxia, no vascular malformation or embolism, and then tracheal tube was removed. The patient was discharged from the hospital 5 days after the procedure. The patient has a good prognosis.

Case 3: a 43-year-old female patient, 55 kg, was admitted 1 month after pancreaticoduodenectomy with 1 day of fever. She was admitted with a diagnosis of intra-abdominal infection and type 2 diabetes mellitus. Due to recurrent vomiting, the physician arranged for her to have a jejunal nutrition tube placed under gastroscopy. On August 3, 2020, the patient underwent gastroscopy under local anesthesia. After confirming the absence of gastric contents, the procedure was subsequently started with 12 mL of intravenous propofol at 16:10, with additional 2 mL of propofol intermittently during the operation. At 16:35, the patient's SpO_2_ suddenly dropped to 76%, and the patient was immediately ventilated with a face mask under pressure, followed by a gradual increase in the patient's oxygen saturation to 89%. During this process the patient's blood pressure fluctuated between 180 to 210/100 to 122 mmHg. At 16:45, the patient's blood pressure suddenly dropped to 64/32 mmHg and heart rate dropped to 30 to 40 bpm. The anesthesiologist instructed the surgeon to immediately withdraw the gastroscope while giving the patient intravenous epinephrine 1 mg and performing tracheal intubation, then the patient was under mechanical ventilation, at that time oxygen saturation rose to 95%, blood pressure rose to 105/58mmHg, ECG showed a heart rate of 62 bpm and E_T_CO_2_ showed 25mmHg. At 17:00 the patient's heart rate dropped to 30 to 40 bpm again and blood pressure and SpO_2_ could not be measured. The anesthesiologist immediately organized to give the patient resuscitation measures such as chest compressions, intermittent intravenous epinephrine, and ice caps. Then the patient's systolic blood pressure fluctuated from 58 to 85 mmHg, heart rate from 30 to 50 bpm, oxygen saturation from 75% to 90%, and E_T_CO_2_ from 8 to 12 mmHg. At 17:17, the anesthesiologist performed a TEE, which showed a significant enlargement of the right heart and a large volume of gas visible in the right and left heart chambers, inferior vena cava, hepatic veins, and pulmonary arteries. The patient was diagnosed with air embolism (Fig. 2 B). Due to hemodynamic instability, the patient received extracorporeal membrane oxygenation for life support treatment. On August 5, the patient developed multi-organ dysfunction, and the family eventually decided to abandon treatment.

## 3 Discussions

The diagnosis of air embolism in case 1 was based on the following:10 minutes after the surgeon injected about 200 mL of air into the ureter, the patient first showed a decrease in E_T_CO_2_, followed by a transient extreme elevation of the ST segment of ECG; TEE showed enlargement of right atrium and right ventricle, left deviation of atrial septum, moderate pulmonary hypertension, and blood gas analysis showed persistently elevated lactate levels when hemodynamics was stable; CT scan showed focal cerebral ischemic-hypoxic injury, and the persistent coma could not be explained by stroke and transient blood pressure changes, and the neurological symptoms of the patients were gradually improved after HBOT on the 4th day after operation. The first presentation of this patient was a sudden decrease in E_T_CO_2_, followed by extreme elevation of the ST segment of ECG. This ECG change is commonly seen in cardiac patients during extracorporeal circulation and cardiac catheterization.^[4]^ The “gold standard” for detection of venous air embolism is TEE due to its ability to detect as little as 0.02 mL/kg of air.^[5]^ The E_T_CO_2_ and ECG are highly sensitive but not very specific. The E_T_CO_2_ decreases, when the patient's blood pressure drops and the pulmonary circulation decreases. This finding also occurs with pulmonary embolism, massive blood loss, circulatory arrest and disconnection from the anesthesia circuit.^[1]^ Myocardial ischemia may be observed ECG changes.^[4]^ After the patient was in the supine position, the transthoracic ultrasound detected the suspicious gas in the cardiac chambers. Because the patient's vital signs were stable after symptomatic treatment, the transthoracic echocardiographic images were unclear and were not stored in time. Forty minutes later, TEE only showed an enlarged right heart with no significant gas. After the operation, the patient awoke and the tracheal tube was routinely removed, ignoring the possibility that air could have entered the left cardiac system and blocked the small arteries, causing impaired oxygenation of the cerebral microcirculation. Due to the lack of a gold standard for direct diagnosis on TEE imaging, the multidisciplinary consultation did not support the diagnosis of air embolism and the patient was not treated with HBOT in the first instance. Although consciousness and muscle strength gradually recovered, the patient still had optic nerve damage. It is reported that when the operator injected 240 mL of air under the PCNL treatment, the patient developed circulatory failure. The pelvic-calvarium system has a 30 mL capacity,^[6]^ and the air injected by the operator exceeded the volume of the renal calyx. The air may enter renal veins from the pelvicalyceal system. Subsequent entry into the right heart will obstruct the pulmonary outflow tract and/or pulmonary.^[6]^ When a large amount of air enters the venous system, it can travel through a pulmonary arteriovenous malformation or cardiac shunt into the arterial system. If enough air enters and overwhelms the lung's filtration system, it can diffuse into the arterial side.^[7]^ The most common intracardiac shunt is a patent foramen ovale.^[8]^ Other shunts, such as atrial or ventricular septal defects, pulmonary shunts and arterio-venous shunts could potentially cause systemic air embolism.^[9]^ Interestingly, septal defects, such as PFOs, persist in up to one-fourth of the general population.^[6]^

Water intoxication is common in hysteroscopic surgery. Case 2 had decreased oxygen saturation, but conventional treatment for water intoxication did not improve significantly, and air embolism was immediately diagnosed by TEE. As TEE suggested gas in the left cardiac system, the patient was not immediately removed from the tracheal tube after awakening, but was promptly treated with HBO. Convulsions occurred during HBOT and symptomatic treatment was given. After 2 times of HBOT, the patient recovered completely. In a study of 5707 procedure of hysteroscopic endometriectomy, 5(0.09%, 1/1140) patients developed severe air embolism and respiratory and circulatory failure.^[10]^ During the hysteroscopic procedure, the patient is in a head-down position with the surgical site above the level of the heart, which can predispose to air embolism. In addition, continuous irrigation and electrocautery can introduce or produce tiny air bubbles. Air can enter the circulatory system through a ruptured vessel or an open venous sinus.

The third case was a patient with pancreatic head cancer, half a month after whipple procedure, who was scheduled to have a jejunal nutrition tube placed. As the operator inserted the gastroscope to begin the procedure, the patient's SpO_2_ dropped and circulatory collapsed. Subsequently, TEE revealed large amounts of gas in both the right and left heart chambers. Despite active resuscitation, the large amount of gas entered the left ventricular system and the patient was unable to receive HBOT during ECMO-assisted life support treatment, which was eventually abandoned by the family. Air embolism can occur not only during surgical procedures such as neurosurgery, obstetrics and gynecology, and urology, but also during non-surgical procedures such as endoscopy, pain management, and blood flow lavage.^[4]^ Air embolism can result from any endoscopic procedure, but is most commonly associated with endoscopic retrograde cholangiopancreatography.^[9]^ Air embolism occurs in nearly 2.5% of endoscopic retrograde cholangiopancreatography patients.^[11]^ But seriously life-threatening air embolism is rare. Although the mechanism by which air is introduced into the blood circulation is debated, but many researchers believe that mucosal damage due to high pressure air, biliary venous fistula and transection of duodenal vein radicles among others may be the underlying mechanism for the introduction of intravascular air.^[9]^

The effect of an air embolus depends upon both the rate and the volume of air introduced into the circulation.^[1]^ The lethal volume has been described as between 200 and 300 mL, or 3 to 5 mL/kg.^[4]^ General anesthesia with tracheal intubation or laryngeal mask, air embolism first manifests as a drop in E_T_CO_2_; in non-intubated general anesthesia, it first manifests as a drop in SpO_2_; While in conscious sedated patients, dyspnea occurs first, followed by a drop in SpO_2_. When a large amount of gas enters the circulatory system, circulatory collapse often occurs and blood pressure drops. When gas enters the left heart causing gas embolism in the arterial system, it may lead to extreme sympathetic excitation and a transient increase in blood pressure. Transient hypertension occurred in case 1 and case 3 in this paper. The mechanism may be related to systemic inflammatory reaction and stress-induced sympathetic overexcitation. CO_2_ can be quickly absorbed and highly dissolved in water or blood.^[10]^ Therefore, the use of CO_2_, but not of air, is strictly recommended to reduce the incidence of emboli.^[12]^ Early diagnosis of air embolism is very important. The most beneficial therapy in eliminating the gas bubbles is HBOT.^[13]^ HBOT can improve tissue oxygenation and reduce the effect of embolic ischemia. Its mechanisms of beneficial action in gas embolism include: reduced bubble volume, increased diffusion gradient out of the bubbles, oxygenation of hypoxemic tissues, amelioration of cerebral edema, reduced platelet aggregation, and activation of the coagulation cascade due to bubble induced endothelial trauma, decreased endothelial binding of leukocytes, and prevention of oxygen-free radical release.^[13,14]^

## 4 Conclusions

In summary, many medical procedures can lead to air embolism, although severe air embolism is rare, once it occurs, it can be life-threatening. Therefore, it is very important to raise awareness of the risk, prompt diagnosis and effective treatment. Once air embolism is considered, echocardiography should be performed immediately, imaging results should be preserved, effective respiratory and circulatory support should be maintained, and hyperbaric oxygen therapy should be given as soon as possible.

## Author contributions

**Conceptualization:** Xueqin Zheng, Guang-rong Duan.

**Formal analysis:** Yue Le, Jian He, Xueqin Zheng.

**Investigation:** Jiling Guo, Xueqin Zheng.

**Writing – original draft:** Jiling Guo, Xueqin Zheng, Hanbing Wang.

**Writing – review & editing:** Jiling Guo, Hong Wang, Jian He, Zhihao Zhang, Hanbing Wang.
